# Multinucleated Giant Cell Formation as a Portal to Chronic Bacterial Infections

**DOI:** 10.3390/microorganisms8111637

**Published:** 2020-10-23

**Authors:** Jacob L. Stockton, Alfredo G. Torres

**Affiliations:** 1 Department of Microbiology and Immunology, University of Texas Medical Branch, Galveston, TX 77555, USA; jlstockt@utmb.edu; 2 Department of Pathology, University of Texas Medical Branch, Galveston, TX 77555, USA

**Keywords:** *Burkholderia pseudomallei*, *Mycobacterium tuberculosis*, multi nucleated giant cell, persistence, chronic infection

## Abstract

This review provides a snapshot of chronic bacterial infections through the lens of *Burkholderia pseudomallei* and detailing its ability to establish multi-nucleated giant cells (MNGC) within the host, potentially leading to the formation of pyogranulomatous lesions. We explore the role of MNGC in melioidosis disease progression and pathology by comparing the similarities and differences of melioidosis to tuberculosis, outline the concerted events in pathogenesis that lead to MNGC formation, discuss the factors that influence MNGC formation, and consider how they fit into clinical findings reported in chronic cases. Finally, we speculate about future models and techniques that can be used to delineate the mechanisms of MNGC formation and function.

## 1. Introduction

Melioidosis is a severe disease caused by the Gram-negative pathogen *Burkholderia pseudomallei* (*Bpm*). *Bpm* is a Category B, Tier 1 select agent, based on the Centers for Disease Control (CDC) classification. The focal point of melioidosis is Southeast Asia and northern Australia. However, increasing surveillance and diagnostic capabilities have revealed that *Bpm* is also found in the soil in the Middle East, sub-Saharan Africa, the Caribbean islands, and the Americas [1]. Recently, a non-travel related case of melioidosis was identified in Texas and, together with another prior case in the same area, suggest that *Bpm* might be present in the soil of the continental United States [2]. It has been estimated that there are 165,000 cases of melioidosis a year with 89,000 deaths globally [1]. Melioidosis can manifest in a variety of clinical presentations [3], giving *Bpm* the nickname “the Great Mimicker” because the disease is easily misdiagnosed. The majority of human melioidosis cases are classified as acute and around 18% result in chronic or latent infections [3]. These latent infections are generally manifested as symptomatic or asymptomatic, leading to abscesses in the liver, spleen, or lung. The severity of the abscess seems to correlate with the number of bacteria taking refuge within the lesion, and have a bias for the spleen and lung; however, liver abscesses have also been observed [4]. These abscesses have been clinically identified as granuloma-like lesions and often are confused with extrapulmonary tuberculosis. Only after diagnostic tests can the *Bpm*-induced lesions be distinguished from *Mycobacterium tuberculosis*, because the histopathology is nearly identical [5,6,7]. One hallmark of *Bpm* infection, both during the acute and chronic stages, is the formation of multinucleated giant cells (MNGCs) [8]. It has been postulated that *Bpm* uses MNGCs as a mechanism to spread from cell-to-cell to evade the external immune system, without fully understanding the host–pathogen-mediated mechanisms of formation. The aim of this mini review is to provide an overview of *Bpm* induced MNGC formation, to discuss factors that influence their development, to consider the role of these lesions in disease, and to highlight the features of *Bpm*-induced MNGCs to MNGCs in chronic tuberculosis (TB) infections. Finally, we discuss future studies to understand the biological relevance of MNGCs in melioidosis.

## 2. *Bpm* Pathogenesis Process Leading to MNGC Formation

Within a mammalian host, *Bpm* is a facultative intracellular pathogen that can invade and survive within nearly all cell types, phagocytic and nonphagocytic alike [9,10]. Whitely et al. demonstrated the ability of *Bpm* and the closely related BSL2 pathogen *Burkholderia thailandensis* to establish an infection in a variety of primary cell lines and noted that the bacteria thrived in bronchial epithelial and vein endothelial cells, suggesting these locations as possible in vivo colonization sites [9]. The intracellular life cycle of *Bpm* can be broken down into three distinct stages: invasion/endosomal escape, cytoplasmic replication/motility, and cell-to-cell spread. 

When invading cells, *Bpm* is taken up within an endosome and by preventing lysosomal fusion, it escapes into the cytoplasm in a type 3 secretion system (T3SS)-dependent manner [11]. *Bpm* wields three T3SSs (1, 2, and 3). T3SS-1 and 2 are involved in virulence against plants and share homology with T3SSs found in other plant pathogens [12,13]. T3SS-3 is primarily involved in mammalian invasion and has been named the *Burkholderia* secretion apparatus (Bsa) [11]. T3SS-3 mutants exhibit decreased invasiveness and partial attenuation in vivo [14]. Another study of T3SS-3 demonstrated that *Bpm* was severely delayed in escaping the phagosome when T3SS-3 was inactive [15]. The exact mechanism of endosomal escape has yet to be elucidated but effectors from T3SS-3 have been implicated in later pathogenesis events, such as regulation of both actin-based motility and MNGC formation [16]. 

The second stage of the intracellular life cycle involves *Bpm* replicating within the cytoplasm and the mobilization of host actin, while evading and/or subverting the bactericidal pathways being activated within the host cell. A recent study [17] demonstrated that *Bpm* upregulates genes associated with combating oxidative stress once free in the cytoplasm. This agrees with another study [18], that showed that *Bpm* induces host factor heme oxygenase 1 (HO-1), which corrals Reactive Oxygen Species (ROS) and promotes host cell survival. Addition of a HO-1 inhibitor resulted in decreased bacterial burdens and an increase in host cell survival. The other occurrence during this stage in the lifecycle is the commandeering of host actin by bacteria to facilitate motility. *Bpm* uses several proteins to accomplish this, but two are vital: BimA and BimC [19,20]. Upon endosomal escape, BimA localizes on one end of the bacterium and oligomerizes to polymerize actin. BimC complexes with BimA but the function of this complex is unknown. Another protein that is involved in the actin polymerization process is BipC, a Bsa effector. Vander Broek et al., showed that BipC can bind both monomeric actin and filamentous actin but is unable to stabilize it [16]. *Bpm bipC* mutants are attenuated in the BALB/c mouse model of melioidosis and exhibited decreased adherence, phagosome escape, and intracellular survival [21].

Upon establishment of actin-based motility, *Bpm* localizes to the plasma membrane and begins to extend the membrane into filopodia-like structures to bring the membrane into proximity with the neighboring cell [16]. This allows for effective engagement of the type 6 secretion system (T6SS), whose activation is dependent on VirA sensing the host cytoplasmic glutathione levels [7]. *Bpm* has six T6SSs encoded in the genome, but T6SS-5 was shown to be needed for pathogenesis of eukaryotic cells [22]. Although T6SSs are commonly used for interbacterial competition and delivery of antibacterial effectors, only *Bpm* T6SS-1 and T6SS-4 seem to have this functionality [23]. *Bpm* intercellular spread via the formation of MNGCs is T6SS-5 dependent. Several studies have demonstrated that individual deletions of essential structural components of T6SS-5 attenuated the infection and abolished cell-to-cell spread [24,25,26,27]. The mechanism for cell fusion and generation of MNGCs is unknown for both the host and the pathogen. The only potential secreted effector molecule that has been identified is VgrG, which is the needle tip protein for all T6SSs [23,28]. The *Bpm* VgrG-5 contains a specialized C-terminal domain (CTD) with effector functionality. When the VgrG-5 CTD is interrupted, cell fusion capability is abrogated. The CTD shares no sequence similarity with proteins of known function so its role in effector function is undetermined [29]. An intact T6SS-5 is necessary for cell fusion but the identity and function of other delivered effector molecules, beyond VgrG-5, remains to be discovered. On the host side, membrane cholesterol and protein content appear to be important for proper membrane fusion, and this is more than likely associated with optimal membrane thickness for T6SS utilization [30]. In another study, MNGC formation was blocked by antibodies that targeted host surface molecules [31]. Taken together, these two studies highlight key host factors that contribute to MNGC formation but also demonstrate the need for further studies. 

## 3. Similarities and Differences between Melioidosis and Tuberculosis

*Mycobacterium tuberculosis* is a historical human pathogen that exhibits a distinct infection cycle and manifestations in the infected host. *M. tuberculosis* is spread via infectious aerosol droplets that are dispersed by the cough of an infected individual. Upon inhalation of the infectious droplets, the bacteria travel to the lower airways where they are internalized by alveolar macrophages and begin to replicate. *M. tuberculosis* accesses the lung parenchyma through an unknown mechanism, but it is hypothesized that infected macrophages migrate through the epithelium, or that *M. tuberculosis* directly infects the epithelial cells and moves deeper into the tissue. Once *M. tuberculosis* has migrated into the tissue, the immune system begins to recruit inflammatory monocytes and leukocytes to contain the infection. This results in the initial stages of granuloma development [32]. The granuloma is a critical structure that has the dual role of containment and safe environment for the pathogen. The factors that influence the outcome of infection are not fully characterized, but it is thought that uncontrolled replication and an imbalance between pro- and anti-inflammatory mediators aids the bacteria to escape containment and result in active TB [33,34]. If the balance is right, the bacteria are contained, and the infection remains asymptomatic with the formation of mature granulomas containing dormant bacteria. The mature granuloma has specialized cells to contain the infection. Some of these include epithelioid macrophages, foam cells, and MNGCs. Epithelioid macrophages have undergone transcriptional changes that make them exhibit epithelial cell characteristics, including a zipper-like morphology [35]. Interruption of the transition results in decreased bacterial burdens [36], suggesting that they contribute to disease by protecting the bacteria from infiltrating leukocytes. This further highlights the duality of the granuloma. The MNGCs that form within the *M. tuberculosis*-derived granuloma have enhanced antigen presentation capabilities, but they exhibit a drastically reduced ability to uptake bacteria [37]. These MNGC are macrophages that became polyploid through interrupted cell division, not cell-to-cell fusion events like *Bpm*-induced MNGC. *M. tuberculosis* has no ability to fuse cells, and continuous inflammatory stimuli causes DNA damage that promotes polyploidy through incomplete cell division [38]. Other studies have shown that IL-4 and IL-13 play a role in cell fusion. The extent of these changes is unclear, but it is thought that the macrophage’s underlying activation state contributes to the response to IL-4 and -13 [39].

There are many parallels between melioidosis and tuberculosis infections, which has led to misdiagnosis in instances where proper diagnostic methods were not applied or available. Some of these parallels include an aggressive, difficult to treat pulmonary or extra-pulmonary disease, in addition to an asymptomatic infection that can reactivate decades later. Both chronic infections tend to result in granuloma-like structures that are often confused with each other; however, because tuberculosis disease is more prevalent globally, it is the usual diagnosis [5]. *Bpm* and *M. tuberculosis* are both intracellular pathogens with a propensity for using macrophages as a replicative niche, and a trojan horse to disseminate to other regions of the body. The risk factors for infection are shared between the two diseases; however, HIV infection is the largest risk group for TB, but the relationship between *Bpm* and HIV co-infection is less understood [40]. *M. tuberculosis* has very predictable manifestations with primarily pulmonary involvement and, in certain cases, dissemination resulting in extrapulmonary tuberculosis. *Bpm* can cause disease in any tissue, that creating a diverse profile of signs and symptoms, and making diagnosis very difficult. 

## 4. Role of MNGCs in Disease

The formation of MNGCs is a feature characteristic of *Bpm* and the closely related bacteria, *Burkholderia mallei* and *B. thailandensis*. MNGCs provide the bacteria with a safe environment to replicate with an abundance of nutrients and resources. The role of MNGCs in whole organism virulence is unclear but the attenuation of T6SS-5 mutants indicates this secretion system is critical to *Bpm* infection [25,26,28]. *Bpm* can form large and heterogenous MNGCs composed of macrophages and neutrophils. The inclusion of epithelial and endothelial cells in heterogenous MNGCs has not been demonstrated, but it is potentially possible based on in vitro studies [9]. MNGCs have been found within the granuloma-like lesions formed during chronic infections, suggesting a larger role in long term colonization [7]. A hypothesis that we have been investigating is that MNGCs are initially a sanctuary for *Bpm* and then act as a nucleation point for the generation of a granuloma-like structure. Factors that influence the switch between acute infection and chronic colonization are largely unknown, but the induction of toxin-antitoxin systems has been implicated in the generation of metabolically dormant persister cells that cause latent infection [41]. *Bpm* infection induces cell death; the structural components of T3SS have been shown to be potent inducers of caspase-1-dependent IL-1β secretion and pyroptosis in murine macrophages [42]. During pyroptosis, the intracellular niche is destroyed, and bacteria are exposed to the external immune system; however, *Bpm* upregulates cytoprotective host factors to preserve the integrity of the intracellular environment [18]. Alternatively, it has been suggested that the host response to MNGC formation centers around type 1 IFN. Ku et al. demonstrated that cell fusion acts as a damage-associated molecular pattern (DAMP), triggering the cGAS-STING pathway that leads to autophagic cell death [34]. The presence of bacterial effectors that manipulate cell death pathways has not been established but it is likely that they exist in some capacity, based on the successful intracellular lifestyle of *Bpm* and the wide array of weaponry the pathogen uses within the cell [43]. The shift from active to persister bacteria would shut down the cytoprotection and manipulation exhibited by *Bpm* and cause the MNGC to undergo cell death, creating foci of necrosis and inflammation, similar to those seen at the center of the mature granuloma-like lesions of chronic melioidosis patients. 

**Figure 1:**
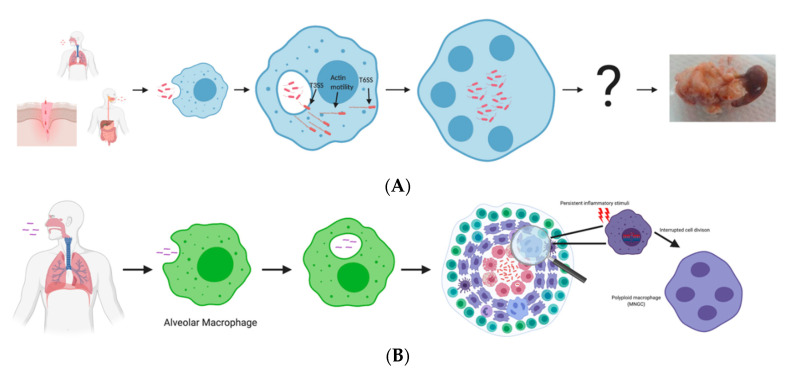
Comparison of pathogenesis events in *Burkholderia pseudomallei* (*Bpm*) and *Mycobacterium tuberculosis*. *Bpm* (**A**) enters the host through inhalation, ingestion, or percutaneous routes. Once in the host, it enters cells and escapes the endosome/phagosome via T3SS activity and becomes free in the cytoplasm. Hijacking host actin, *Bpm* moves to the perimeter of the cell to engage T6SS. Using T6SS activity, *Bpm* fuses neighboring cells and forms MNGCs and, eventually, these can establish granuloma-like abscesses through an unknown mechanism. *M. tuberculosis* (**B**) is inhaled, where it travels into the airways and encounters alveolar macrophages. Once internalized, *M. tuberculosis* prevents phagolysosomal fusion and proliferates in the phagosome. Alveolar macrophages laden with bacteria penetrate the epithelium and travel deeper into the tissue, where it triggers the response that results in the formation of the granuloma. The MNGCs found within the granuloma form persistent inflammation, causing DNA damage and atypical cell division, resulting in polyploidy. Scheme of granuloma colored cells: red (macrophages; with and without bacteria, foam, and apoptotic cells), purple (epithelioid macrophages), blue (MNGCs), light purple (neutrophils), dark purple (dendritic cells), turquoise (T-cells), light blue (B-cells), green (Natural Killer cells).

As previously stated, MNGCs have been found in the granuloma-like structures of chronic *Bpm* infections but mostly on the perimeter of the central necrotic core that houses most of the bacteria [4,44]. This is consistent with the granuloma structure in *M. tuberculosis* infection where MNGCs are scattered throughout the periphery of the epithelioid cells, foamy macrophages, and lymphocytes. It is unknown whether these MNGCs were fused by *Bpm* or, like the *M. tuberculosis* granuloma, they have become polyploid due to inflammation-induced DNA damage or cytokine influence [38,39] (Figure 1). Studies have shown that peripheral blood mononuclear cells (PBMCs) exposed to beads coated in *M. tuberculosis* extract begin to form granulomas, including MNGCs, without live bacteria [45]. It is reasonable to speculate that the formation of MNGCs during *M. tuberculosis* infection is a passive process from the bacterial side. This contrasts with *Bpm* infection in which live bacteria and an intact T6SS-5 are required for MNGC formation. It is possible that the MNGCs observed within the granuloma-like structures are sterile environments and have undergone the same polyploid events as those within *M. tuberculosis* granulomas. The MNGCs that were actively formed by *Bpm* were likely at the center and resulted in the necrotic core that is full of extracellular and intracellular bacteria. This idea is supported by post-mortem or post-surgical histopathology of infected spleens, in which patients that died of acute melioidosis exhibited higher amounts of MNGCs compared to chronically infected patients who had organs removed surgically to find granuloma-like lesions. The latter were positive for *Bpm*, and negative for *M. tuberculosis* and fungal diseases that could cause the formation of the granuloma [7]. Conejero et al. developed an animal model for chronic melioidosis and found that multiple and distinct types of lesions occur. Two of these include necrotic and non-necrotic granulomas. The necrotic granulomas are characterized by a caseous core surrounded by a distinct fibroblast and epithelioid macrophage layer that separates the necrotic from the non-necrotic area. The non-necrotic granulomas are comprised of mostly epithelioid macrophages, lymphocytes, and scattered MNGCs [46]. This study demonstrated that caseous granulomas are formed during chronic melioidosis, but the response is heterogenous, and also includes non-necrotic granulomas and pyogranulomatous lesions. The severity of the lesion, both microscopically and macroscopically, correlates with the number of bacteria within the lesion, which agrees with more recent studies [4]. Based on comparisons between laboratory and clinical studies, it is evident that MNGCs are the keystone event of *Bpm* pathogenesis. However, many more studies are needed to delineate the mechanisms of their formation and define the role they play in chronic infection.

## 5. Future Directions and Models

It is becoming clear across all disciplines that physiology happens in three dimensions (3D), and many responses are dependent on cells interacting with other cells and/or the extracellular matrix (ECM) proteins in a spatiotemporal manner. Many common cell culture techniques fail to faithfully recapitulate the responses that occur in vivo because they lack the 3D interactions with the cellular environment [47]. That is not to say that these methods are invalid but the translational power behind them is limited compared to novel 3D cell culture systems being developed. The implications of 3D technologies for a wide array of cellular responses have been recently reviewed [48], and this review indicates that interactions that occur in 3D have a drastic effect on the nature of the responses. For example, in 2D cultures necrotic cells detach and float into the media, whereas in vivo and in 3D cultures necrotic cells are trapped, and the surrounding cells are forced to interact with the dying cell. With the complex nature of *Bpm* pathogenesis culminating in cell fusion events and granuloma-like lesion formation in chronic cases, it would be very useful to adapt some of the advanced cell culture techniques to study MNGCs and granuloma formation in systems that more closely resemble the organs where the events take place. The tuberculosis field has made significant progress in this area and many novel in vitro techniques have been used to define the role of these cell structures in the pathology of *M. tuberculosis* [49]. Because of the similarities between melioidosis and tuberculosis, the adoption of these methods to study melioidosis is attractive but limited in scope because the primary focus is the lung. Organoids are simplified, miniature versions of tissue that are generated in vitro but still have characteristics found in vivo. Organoids represent an attractive situational alternative to in vivo and in vitro modeling due to the levels of customization, complexity, and control. *Bpm* can colonize and cause disease in most tissues of the body, so expansion of the repertoire of organoid models would be needed to characterize the behavior of *Bpm* in different organs and systems. Fortunately, many of these organ models are already in development or in use in other areas of biomedical research and can be modified to accommodate *Bpm* studies [50,51,52]. 

The most attractive organoid models for *Bpm* are those recapitulating lung, liver, and spleen, and that can be used to study the dynamics of chronic infection and granuloma formation. Conejero et al. [46] demonstrated that different chronic colonization sites result in distinct types of lesions and bacterial loads, but the in vivo study was limited to mostly histopathology. Studying this in an organoid model for each site would offer the ability to more closely examine the host–pathogen interactions. An in vitro system that also mimics the microanatomy would greatly increase the ability to study specific contributions from each type of immune cell, transiently monitor the activity and transformation of different cell populations and improve imaging of the system by customizing cell lines specifically for imaging. The use of organoids would help to decipher if certain events seen in vitro are artifacts or biologically relevant. An example would be the formation of heterogenous MNGCs; *Bpm* can fuse macrophages and neutrophils in co-culture [9], but the in vivo occurrence has yet to be seen. An organoid system might not be able to completely replicate the conditions in vivo but offers advantages over both standard in vivo and in vitro techniques in certain situations. Another interesting avenue for organoid development could be the development of a model for neurological melioidosis. Central nervous system (CNS) involvement is relatively rare in human melioidosis but more common in chronic murine models. It has been demonstrated that *Bpm* invades the olfactory bulb and can fuse glial cells [10]. Micro-abscesses in the white matter and thickening of the trigeminal nerve has been observed in human patients with CNS melioidosis, suggesting that *Bpm* invades the CNS through axon transport and bypassing the blood–brain barrier [53]. The BimA protein is important for neuro-invasion, most likely because the bacteria need to travel long distances up the axon and to spread to adjacent cells [54]. Organoid modelling of the spinal cord would provide a unique opportunity to explore *Bpm*’s ability to traverse long distances through cell-to-cell spread.

Another modeling system that shows potential for studying MNGCs and granuloma formation is the zebrafish. The zebrafish is commonly used with *Mycobacterium marinum*, which is a surrogate organism for *M. tuberculosis*, as a model to study granuloma formation. The granuloma structures formed in the zebrafish model of *M. marinum* are nearly identical to those formed by *M. tuberculosis* [55]. This model is attractive for many reasons; one is the ability to examine the contributions of innate and adaptive immune components separately because the embryonic stage of the zebrafish only possesses an innate system. Another attractive characteristic is the growing number of genetic knockout strains of zebrafish, including important immune mediators [34]. This model has been used as a bridge between in vitro models and rodents to test the efficacy of therapeutics with a high degree of success. The zebrafish has been used to study *Burkholderia cepacia* complex (Bcc) therapeutics and pathogenesis [56,57]. However, establishing a zebrafish model for *Bpm* infection has two immediate obstacles: biosafety concerns that arise due to the infection of an aquatic animal with a tier 1 select agent and, second, the possibility that *Bpm* might be too virulent for the model. Both obstacles can be easily overcome by using *B. thailandensis*, which is used as a BSL2 surrogate for *Bpm* and exhibits many of the same characteristics of infection, including MNGC formation. The zebrafish has been used to assess virulence in *B. thailandensis* mutants but has not been used to explore MNGC formation or chronic infections [39]. Zebrafish offer an alternative to traditional in vivo and in vitro models much in the same way as organoids. Some of the characteristics include a vertebrate immune system, ability to separate innate and adaptive components of the immune system, translucent skin for easy real-time imaging, and genetic knockout fish strains. All of these characteristics are useful for the study of *Bpm* and bridging the gap between simple cell culture and murine models.

## 6. Final Remarks

The goal of this mini review was to shed light on MNGC formation and function during *Bpm* infection. MNGCs are linked to the function of *Bpm* T6SS-5 and interruption results in attenuation in murine melioidosis. We propose that MNGCs play a critical role in infection and granulomatous lesion formation. More work is needed to understand MNGCs formed by *Bpm* and how they compare to MNGCs that form within *M. tuberculosis* granulomas. To gain further insight, we strongly believe that adapting 3D cell culture methods to closely mimic the microenvironment within the host will provide useful information. As indicated, these types of organoid methods, in addition to the zebrafish, have been widely used within the *M. tuberculosis* field, which makes commandeering them an attractive option for *Bpm* studies. Overall, the MNGC is an understudied area of *Bpm* but has proved to be difficult and, therefore, innovation is a necessity to begin unraveling the mysteries within.
